# A Review of CO_2_ Sequestration Projects and Application in China

**DOI:** 10.1155/2014/381854

**Published:** 2014-07-01

**Authors:** Yong Tang, Ruizhi Yang, Xiaoqiang Bian

**Affiliations:** The State Key Laboratory of Oil & Gas Reservoir Geology and Exploitation Engineering, Southwest Petroleum University, Chengdu 610500, China

## Abstract

In 2008, the top CO_2_ emitters were China, United States, and European Union. The rapid growing economy and the heavy reliance on coal in China give rise to the continued growth of CO_2_ emission, deterioration of anthropogenic climate change, and urgent need of new technologies. Carbon Capture and sequestration is one of the effective ways to provide reduction of CO_2_ emission and mitigation of pollution. Coal-fired power plants are the focus of CO_2_ source supply due to their excessive emission and the energy structure in China. And over 80% of the large CO_2_ sources are located nearby storage reservoirs. In China, the CO_2_ storage potential capacity is of about 3.6 × 10^9^ t for all onshore oilfields; 30.483 × 10^9^ t for major gas fields between 900 m and 3500 m of depth; 143.505 × 10^9^ t for saline aquifers; and 142.67 × 10^9^ t for coal beds. On the other hand, planation, soil carbon sequestration, and CH_4_–CO_2_ reforming also contribute a lot to carbon sequestration. This paper illustrates some main situations about CO_2_ sequestration applications in China with the demonstration of several projects regarding different ways of storage. It is concluded that China possesses immense potential and promising future of CO_2_ sequestration.

## 1. Introduction

The enormous emission from greenhouse gas, predominated by CO_2_, has caused increasing threat to human environment and the ecological system. The current global annual carbon emission reaches up to more than 30 billion tons. In China, fossil fuel takes up 92.6% of the total energy; 67.1% of CO_2_ is generated from coal and petroleum. Moreover, China is the biggest CO_2_ emitter by now. According to International Environment Agency, emission from China would overpass the whole world's CO_2_ emission by 2020 [1]. Therefore, it is an urgent requirement for China to transform from high-carbon to low-carbon society.

According to “Report on the Development of Low Carbon Economy of China (2012),” China is the largest country for carbon emission reduction. The world's largest carbon emission reduction project started in China in 2005, which is expected to reduce about 19 million tons of CO_2_ equivalent emission every year [2]. 1.5 billion tons of CO_2_ emission has been reduced during “11th five-year plan” in China, and it is likely to cut 7 billion tons of CO_2_ in 2020.

Various ways of reducing carbon emission have already been applied in China. And, among them, a major mitigation method is carbon capture and sequestration (CCS).

It is believed that CCS is the long-term isolation of carbon dioxide from the atmosphere through physical, chemical, biological, or engineered process. It includes carbon sequestration through forestation, soil carbon sequestration, direct ocean injection of CO_2_ either into the deep seafloor or into the intermediate depths, and the deep geological sequestration, or even direct conversion of CO_2_ to carbonate minerals [3], of which geological sequestration is a major component. CCS is an effective way for China to alleviate pollution and enhance the oil recovery, and most underground spaces in China are good for CO_2_ geological storage [4]. However, CCS has just started in China, and there is a certain gap between China and abroad. But there are still some technical foundations in China, especially in the area of CO_2_ recycling and injection [5].

Several main types of geological storage media for carbon sequestration are mostly considered in China: depleted or active oil and natural gas field, coal layers, and deep saline aquifers. The win-win effects make oil and natural gas field and coal layers are the promising storage media with great advantages. By using CO_2_ for oil and gas fields and the coal seams, CO_2_ is stored and the production is increased. And the deep saline aquifers are attractive due to the large storage capacity of interest [6–9].

**Figure 1:**
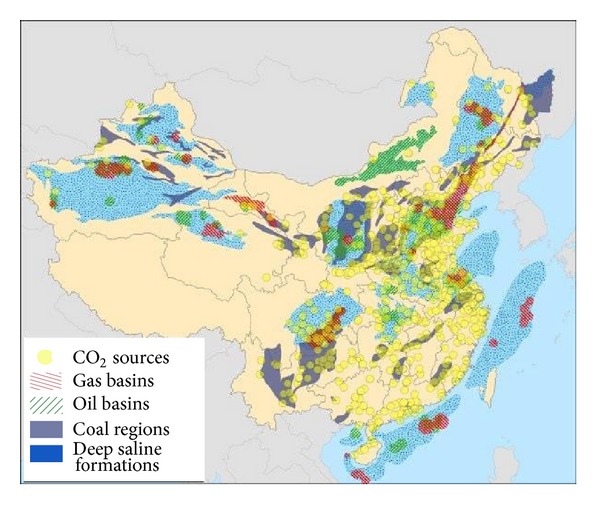
Locations of large CO_2_ point sources and CO_2_ storage reservoir in China (from Dahowski et al. [10]).

Figure 1 shows a map of large (100+ kt CO_2_/yr) CO_2_ sources and potential candidates for geologic CO_2_ storage basins in China [10].

## 2. CO_2_ Source Supply

A large amount of CO_2_ emitted by industry could be supposed to serve as the significant potential CO_2_ source to meet the storage demand if only the advanced capturing technology is available. And coal-fired power plant is the focus of CO_2_ capture due to its excessive emission and the energy structure in China [3, 11, 12]. Therein, technologies of solvents method, membranes separation, solid sorbents, and cryogenic fractionation have been applied to separate CO_2_ from natural gas or waste gas [13]. CO_2_ could be transported via highway, railway, shipping, and pipeline, of which pipeline is especially suitable for large-scaled and long-term gas injection, like the CO_2_-EOR project in Jilin oilfield.

Many efforts have been used to develop more efficient techniques for CO_2_ capture in China, like the blended solvent presented by the Joint International Center for CO_2_ Capture and Storage of Hunan University, MSA chemical absorption technique developed by Sinopec. And the research of Joint Research Center for Advanced Environmental Technology of Tsinghua University showed that carbon-based materials have high adsorption capacity with merits of low cost and easy regeneration. And Fang indicated that membrane vacuum regeneration has the potential to reduce energy consumption greatly [14].

CO_2_ capturing projects have been progressing extraordinarily throughout China. Post-Combustion Capture CO_2_ and Refining Utilization project with capacity of 0.12 × 10^6^ t/yr in China is the biggest postcombustion capture project in the world then [15]. Sinopec has built the 100 t/d CCUS (Carbon Capture, Utilization and Storage) project on coal-fired power plant flue gas and deployed three ways of recycling CO_2_ with more than 80% of capture efficiency and over 95% of purity. China Huaneng Group has built the first coal-fired power plant CO_2_ capture demonstration project in 2008 with 3000 t/yr of CO_2_ capture ability and completed the second power plant in Shanghai Shidongkou demonstration project with 0.1 × 10^6^ t/yr of CO_2_ capture ability. Shenhua Group launched China's first CO_2_ capture and geologic storage full process demonstration project in 2010 [16]. Moreover, the project with the scale of 50000 t/yr capture capacity which has product purity of more than 99.5% has been put into use in 2012 in Yanchang. And for the future, improving efficiency and reducing cost are the crucial development tendency.

## 3. CO_2_ Sequestration

### 3.1. Estimation of CO_2_ Sequestration Capacity

Several methods have been developed to assess the CO_2_ storage capacity in geological media at home and abroad [17–27]. Examples are listed as follows.

Zhang et al. [18] developed the formula which considers the different storage mechanisms:

(1) $$\begin{array}{c} M_{{\text{CO}}_{2}}=M_{1}+M_{2}+M_{3}+M_{4}, \end{array}$$

where *M*_1_ is the storage capacity of CO_2_ taking the volume previously occupied by produced oil; *M*_2_ is the storage capacity of CO_2_ dissolved in residual oil; *M*_3_ is the storage capacity of CO_2_ dissolved in water contained in reservoir; and *M*_4_ is the storage capacity of CO_2_ reacting with reservoir rock.

Sun and Chen [19] proposed the study to calculate increased oil production and the CO_2_ storage capacity in oil reservoir and depleted oil reservoir.

Proportion of increased oil by CO_2_-EOR is as follows:

(2) $$\begin{array}{c} \%\text{EXTRA}=\{\begin{array}{cc} 5.3\% & \left(API\leq 31\right)\\ \left(1.3\times API-35\right)\% & \left(31<API<41\right)\\ 18.3\% & \left(API\geq 41\right), \end{array}\\ {\text{OOIP}}_{e}=\text{OOIP}\times C, \end{array}$$

where OOIP is the original oil in place, Mt; *C* is the contact ratio between oil and CO_2_. OOIP_*e*_ is the amount of oil that can contact with CO_2_, Mt.

The increased oil production and storage capacity are as follows:

(3) $$\begin{array}{c} \text{EOR}={\text{OOIP}}_{e}\times \%\text{EXTRA},\\ {\text{CO}}_{2}=\text{EOR}\times R_{{\text{CO}}_{2}}, \end{array}$$

where EOR is the increased oil production, Mt; CO_2_ is the storage capacity, t or Mt; *R*_CO_2__ is the ratio between the amount of injected CO_2_ and the amount of increased oil, t/bbl or t/t.

However, for the CO_2_ storage capacity in depleted oil reservoir,

(4) $$\begin{array}{c} {\text{CO}}_{2}=\text{OOIP}\times \text{RF}\_\text{O}\times \text{FVF}\_\text{O}\times \rho {\text{CO}}_{2}, \end{array}$$

where RF_O is the oil recovery when depleted; FVF_O is the formation volume factor; *ρ*CO_2_ is the density of SCCO_2_ under the reservoir temperature and pressure, Mt/m^3^.

Tanaka and coworkers [20, 21] set up two models based on underground structures: model (5) is suitable for aquifers that are well sealed by cap rocks and model (6) for aquifers in monoclonal structures and there may be problem of CO_2_ leakage into the upper portion. Consider

(5) MCO2=Ef×A×h×Φ×ρ×[SgBg(CO2)+(1−Sg)Rs(CO2)],

(6) $$\begin{array}{c} {M\text{CO}}_{2}=Sf\times A\times h\times \Phi \times Rs\left({\text{CO}}_{2}\right)\times \rho , \end{array}$$

where *Ef* is the sweep efficiency (fraction, dimensionless), *A* is storage area (m^2^), *h* is effective formation thickness (m), Φ is effective reservoir porosity (fraction, dimensionless), *Sg* is saturation of supercritical CO_2_ (fraction, dimensionless), *Bg*(CO_2_) is CO_2_ formation volume factor (m^2^/m^3^, reservoir volume/standard volume), *Rs*(CO_2_) is CO_2_ solubility in formation water (m^3^/m^2^), *ρ* is density of CO_2_ at standard condition (kg/m^3^), and *Sf* is the storage factor (fraction, dimensionless).

### 3.2. Geological Sequestration

CO_2_ can be more effectively sequestrated at pressure higher than 7.38 MPa (equivalent depth of about 800 m), and at temperature above 31.1°C, where CO_2_ will stay in a supercritical state with an elevated density up to 600 kg/m^3^, 400 times more condensed compared to that at atmospheric conditions. SCCO_2_ (supercritical CO_2_) is characterized by stable and inert chemical property. Consequently, at pressures and temperatures typically encountered in the field, CO_2_ will behave as a supercritical fluid [28].

CO_2_ geosequestration has been implemented successfully around the world like CO_2_-EOR and storage in Weyburn project of Canada in 2000 [29, 30]; CO_2_ storage in K12-B gas field of The Netherlands in 2004 [31]; the upcoming ROAD project in 2015 with CO_2_ storage in P18-4 depleted gas field of The Netherlands [32]; associated CO_2_ separation and injection into the saline aquifer in Sleipner project of Norway in 1996 [33]; CO_2_ storage in In Salah aquifer of Algeria in 2004 and Snohvit aquifer of Norway in 2008 [34, 35]; CO_2_-enhanced coal bed methane (CO_2_-ECBM) and storage in San Juan Basin of New Mexico in 1995 [36], and other CO_2_-ECBM projects in USA [37, 38].

Research results suggest that CCS can provide a valuable greenhouse gas mitigation option for most regions and industrial sectors in China and can be able to store more than 80% of emissions from these large CO_2_ sources (2900 million tons of CO_2_ annually) at costs less than $70/t CO_2_ for perhaps a century or more [10]. Similarly, various geosequestration projects have been in progress in China, regarding the storage in oil and gas fields, in saline aquifer and in coal seams.

#### 3.2.1. CO_2_ Sequestration in Oil and Gas Field

Carbon sequestration with enhanced oil recovery (CSEOR) is a kind of win-win process to increase oil production and store CO_2_. Moreover, the revenue created could be able to offset the storage cost and bring valuable profit.

CO_2_ has been widely used for EOR around the world. CO_2_-EOR projects now produce about 0.35 × 10^6^ bbls/day in USA, accounting for 5.6% of total USA oil and gas production, compared to just 0.19 × 10^6^ bbls/day in 2000. And approximately 50 million metric tons of CO_2_ is used each year for EOR in USA [39, 40].

CSEOR or CSEGR has been assessed and applied for several oil and gas fields across China. When the buried depth is more than 800 m (guarantee the supercritical state of CO_2_); the CO_2_ storage potential capacity is of about 3.6 × 10^9^ t, assuming that all onshore oilfields in China are used for CO_2_-EOR, and it can reach up to 4.6 × 10^9^ t while considering all onshore oilfields as depleted reservoirs. Therein, reservoirs in northeast and north China have tremendous sequestration potential, accounting for more than 60% of the total capacity [24].

Considering the depth between 900 m and 3500 m, China's major gas fields are able to provide storage capacity of about 30.483 × 10^9^ t of CO_2_, and the proven natural gas resources correspond to storage capacity of 4.103 × 10^9^ t CO_2_. However, gas industry has been started late in China, and there will be no large-scale depleted gas field for a long time. In this way, gas fields should not be used to store CO_2_ in the near future but should serve as the strategic energy reserves due to the good sealing property of depleted gas fields [25].

Oil reservoirs are screened on the basis of oil gravity, reservoir temperature and pressure, MMP, and remaining oil saturation, to determine their suitability for CO_2_ flooding [17]. And several different types of screening criteria have been proposed at home and abroad for CO_2_-EOR and storage [17, 41–44], regarding crude oil properties, reservoir characters, cap formation characters, and economic and environmental issues.

**Figure 2:**
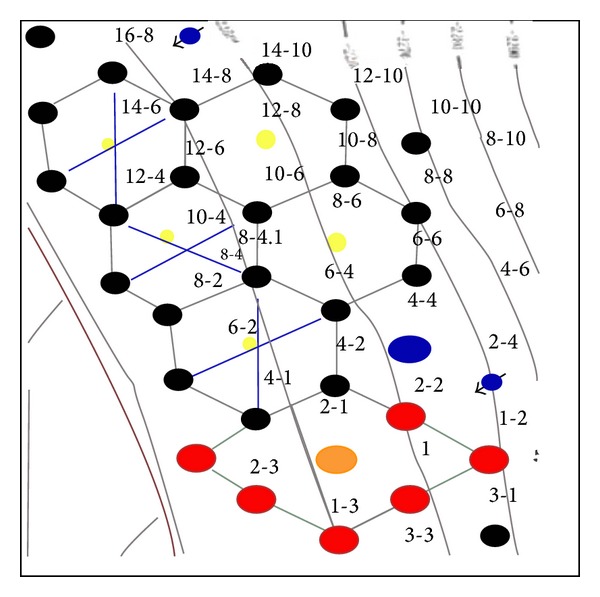
Diagram of well location and surface layout of cross-well seismic lines: yellow dots are CO_2_ injectors, and the seismic lines are in deep blue color (from Ren et al. [45]).

Jilin oilfield, located in northeast of China, is conducting the first large-scale demonstration project on CO_2_-EOR and storage. The oil-bearing formations are characterized by good development of sandbody, good connectivity, and well-defined cap rocks [45]. Natural source of CO_2_ is mainly from natural gas. And miscible flooding can be achieved in block Hei-59 and Hei-79; well location is indicated in Figure 2.

**Figure 3:**
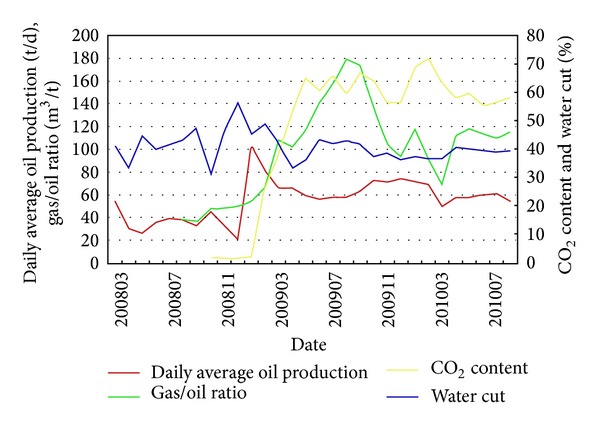
Measured oil production, water cut, CO_2_ content and GOR in the CO_2_ miscible pilot area of Jinlin oilfield (from Ren et al. [45]).

In 2008, Jilin oilfield built a pilot demonstration area of CO_2_ flooding and storage in the Daqingzi oilfield. And in 2009, a demonstration area with its annual CO_2_ storage of 0.2 × 10^6^ t and annual oil displacement of 0.1 × 10^6^ t was established, which indicated realization of commercial application of such technology. Good production response has been observed after about 6 months of CO_2_ injection since April 2008, as shown in Figure 3. Oil production in the whole pilot area has rapidly increased from 20 t/d to around 100 t/d and has been maintained at 60 t/d in 2011. By the end of May 8, 2011, about 0.167 × 10^6^ t of CO_2_ was stored without obvious CO_2_ leakage; and 0.119 × 10^6^ t of oil was produced by CO_2_-EOR. At the same time, a plant was built in the Jilin oilfield to separate and capture 0.2 × 10^6^ t of CO_2_ annually [46, 47]. 0.27 × 10^6^ t of CO_2_ has been safely stored until August 2012 with remarkable economic benefit, with 1 : 1.37 as the input and output ratio [48]. It is expected that, by 2015, the first production area will be built in China, with an annual CO_2_ displacement amount reaching 0.5 × 10^6^ t and an annual CO_2_ storage over 0.7 × 10^6^ t, all of which are equivalent to the total amount of CO_2_ released from burning of 0.3 × 10^6^ t of coal [46].

And the further work will be focused on optimizing EOR performance, verifying of the geocapacity storage in the targeted zones and carrying forward the monitoring programs [45].

**Figure 4:**
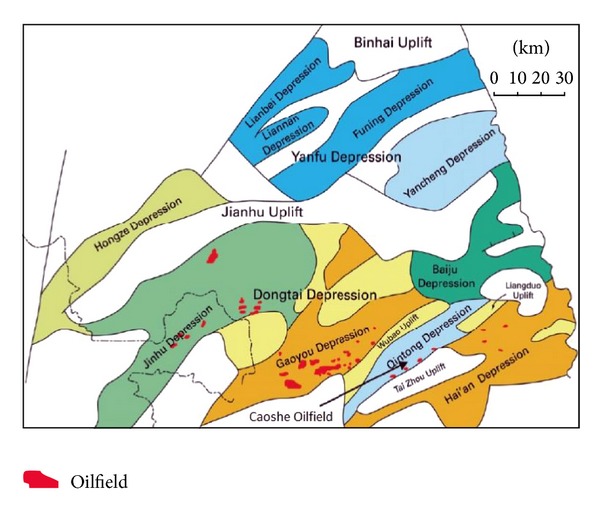
Geotectonic map showing the main depression and uplift regions in the Suibei basin, where the Caoshe oilfield is located (from Zhang [52]).

Caoshe oilfield is located in Subei Basin and has been selected to implement CO_2_-EOR and storage demonstration project. The geological map is shown in Figure 4. Taizhou formation is the main oil-bearing formation in Caoshe oilfield. And during the development periods, the oilfield has developed a complete well pattern of injectors and producers with good well connection, the water cut at the producer has been relatively low, and the reservoir pressure has been well maintained [23, 49].

**Figure 5:**
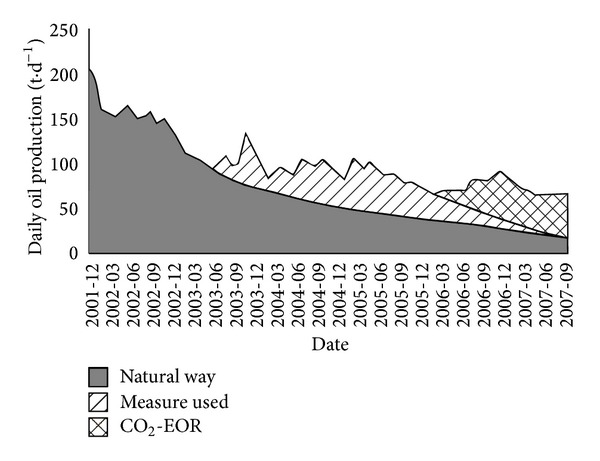
Simulation result of CO_2_ miscible flooding of the Taizhou Formation reservoir in the Caoshe oilfield (from Yu et al. [51]).

Taizhou formation is geologically suitable for CSEOR. Taizhou formation has carried out the CO_2_-EOR pilot test in July 2005, and 5.842 × 10^7^ m^3^ CO_2_ has been injected from July 2005 to December 2009 with increased oil production of 0.03 × 10^6^ t [49, 50]. CO_2_ can achieve a miscible displacement process and be stored safely in the stratigraphic and structure traps of Taizhou formation reservoir [51]. Besides, Nanjing Chemical plant, a synthetic ammonia plant 120 km away from the Caoshe oilfield, would provide a low-cost CO_2_ source for the CCS demonstration project. The detailed numerical reservoir model indicates that the maximum CO_2_ storage capacity at standard condition is estimated to be 0.309 × 10^9^ m^3^.  Figure 5 shows the simulation result of CO_2_ miscible flooding. Furthermore, the revenue from incremental oil production is significant, which cannot only offset the cost of the CO_2_ storage, but also can generate certain economic benefit to Caoshe oilfield [23], while Zhang indicated that the storage cost of CO_2_-EOR process is $25.78/t, based on the economic evaluation model established [52].

The Ordos Basin is the second largest sedimentary basin in China, which takes account for 43% of resources of the whole country. In 2011, the oil and gas production exceeded 0.052 × 10^9^ t of oil equivalents [53]. Ordos Basin is able to provide a huge potential capacity for CO_2_ storage.

Jingbian field is located in central Ordos Basin in northern Shaanxi slope and has been screened out to conduct the CO_2_ sequestration. CO_2_ will be captured from the energy and chemical engineering industrial zone in Jingbian City which is 30 km away from the operation site. And it is estimated to inject CO_2_ of 0.04 × 10^6^ t/yr and increase oil production of 0.05 × 10^6^ t/yr from CO_2_-EOR [53].

Furthermore, various feasibility studies of geological CO_2_ sequestration have been implemented for wide areas of Ordos Basin [54–56]. For example, research indicates that, for 261 production layers of Changqing oilfield, total oil production increment and CO_2_ sequestration amount can reach about 0.098 × 10^9^ t and about 0.239 × 10^9^ t, respectively [54]. Results from the 50-year injection simulation indicate that a total of 450 Mt of CO_2_ can be injected into the targeted reservoir of Majiagou formation (northern Ordos Basin), while 166 Mt of original pore fluids will be displaced by CO_2_ [55].

Additionally, other fields around the country also show good results for CO_2_ application. Xinjiang oilfield, a vital oilfield in western China, is located in Junggar Basin. Around 0.181 × 10^9^ t additional oil could be produced for the total screened out 275 production units, which could provide about 0.495 × 10^9^ t for CO_2_ sequestration capacity [57]. Many mature oil reservoirs in Shengli oilfield (north China) are close to the main CO_2_ sources and have good geographical and geological conditions for CO_2_ storage. The total EOR potential can be 9.997 × 10^6^ t, and the CO_2_ storage capacity can reach 95.539 × 10^6^ t [44]. Zhongyuan oilfield (central China) and Daqing oilfield (northeast China) get obvious recovery increment after CO_2_ flooding.

The associated CO_2_ from natural gas is another major carbon emission. IPCC estimated that about 50 million tons of reservoir-CO_2_ is liberated into the atmosphere every year, from natural gas production [11]. Projecting this to year 2030, and assuming sourness does not increase, the emissions figure could be 150 Mt/yr [58]. And in South China Sea, the geological reserve of CO_2_ is huge [59].

DF1-1 gas field is located in the west of the South China Sea, which is associated with a high concentration of CO_2_. A demonstrative project of CO_2_ sequestration is considered for nearly abandoned southeast block of the lower Group II formation in the DF1-1 gas field, which was reassessed for the safety of CO_2_ storage [58]. The separated CO_2_ would be injected back into the original gas reservoir, similar to the demonstration projects carried out in K12-B (Netherlands).

The feasibility studies showed that the faults in gas field are characteristic of good sealing property for the targeted block. The injected CO_2_ of the southeast block will be effectively trapped in the reservoir because of its good sealing mechanism and poor connectivity with other blocks [60]. Simulation results indicate that CO_2_ can be injected steadily at a rate of 0.140 × 10^6^ Sm^3^/d over 10 years, and the cumulative CO_2_ gas injection can be 0.511 × 10^9^ Sm^3^ for the pressure control required. Zhang et al. [60] showed that unit storage of CO_2_ is approximately $20/t at the current economic situation, while there will be no extra finial returns for this demonstration CO_2_ sequestration project.

On the other hand, CO_2_ injection into oil and gas reservoirs associated with large aquifers takes advantages of lower geological leakage risk from oil and gas traps and large storage capacity from the connected aquifers [61]. Results of cases studies of five oil reservoirs selected from Shengli and Jiangsu oilfields in China demonstrate that CO_2_ storage capacity can be greatly increased if the lateral and underlying aquifers are included.

#### 3.2.2. CO_2_ Sequestration in Saline Aquifer

Deep saline aquifers have proven to be the promising geological media for CO_2_ sequestration due to the large storage capacity and wide availability. The injected CO_2_ can be sequestrated in deep saline aquifers through a combination of physical and chemical trapping mechanisms, which include stratigraphic or structure trapping, residual trapping, solubility trapping, mineral trapping and hydrodynamic trapping [27, 62–64].

143.505 × 10^9^ t CO_2_ can be stored in saline aquifers of China [65]. Most of the north China plain; northern, eastern, and southern Sichuan Basin; southeast of Junggar Basin are the priority for CO_2_ aquifer storage in the future, like the deep saline aquifers in Songliao Basin (northeast China) can contribute about 8.96 × 10^9^ t of CO_2_ sequestration capacity [66].

**Figure 6:**
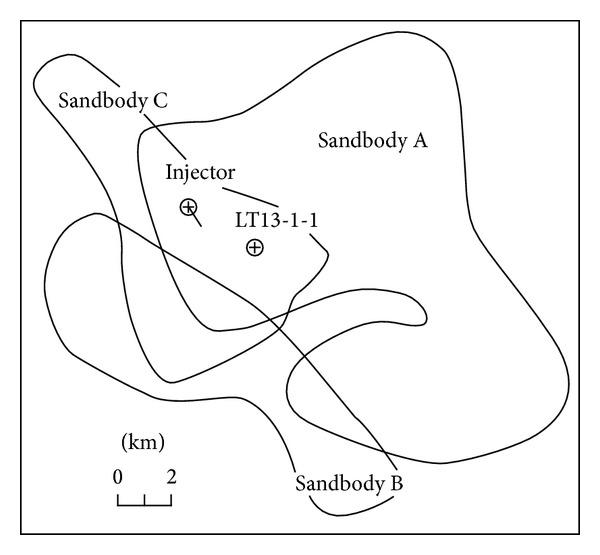
Distribution of sandbodies in the LT13-1 saline aquifer (from Zhang [52]).

**Figure 7:**
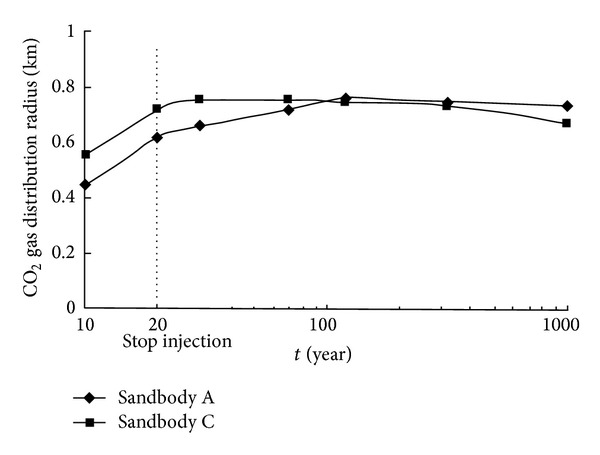
CO_2_ gas distribution radius in sandbodies A and C during and after injection (from Zhang [52]).

Saline aquifer trap LT13-1, located in the east of DF1-1 gas field, 60 km away from the Dongfang gas terminal, has been selected as the target CO_2_ storage site to sequestrate the CO_2_ discharged from the DF1-1 gas terminal [67]. The reservoir is relatively good in homogeneousness and high in salinity, indicating a good trap feature. The injected CO_2_ will be trapped both in a supercritical state and in dissolved state in formation water. Sandbodies A and C of LT13-1 structure can provide a CO_2_ storage capacity of approximately 0.1 × 10^9^ t [67], as shown in Figures 6 and 7. Zhang et al. pointed out that the storage cost is about $33–37/t, slightly higher than abroad due to the high cost of offshore pipeline [68].

Being one of the most typical sedimentary basins in eastern coastal of China, the Bohai Bay Basin is a potential candidate for CO_2_ sequestration. CO_2_ storage in deep saline aquifers is considered as a viable option because of the wide-distribution with a high CO_2_ storage capacity. The CO_2_ storage capacity within the assessing range is 3.9 × 10^9^ t in saline aquifers of Bohai Bay Basin, and storage capacity in Neogene Guantao formation lower than 3500 m is 3.3 × 10^9^ t, accounting for 84.4% of the total potential [70].

Section 3 in the lower part of the Neogene Guantao formation of Beitang Sag, Huanghua depression, near the center of the Bohai Bay Basin, has been chosen as the test site for CO_2_ injection [71]. Due to the good cap-rock layers, CO_2_ can be stored safely in Section 3 in supercritical state. Based on the model (6) proposed by Tanaka, the CO_2_ storage capacity of the Beitang Sag is estimated to be 17.03 Mt.

#### 3.2.3. CO_2_ Sequestration in Coal Seam

China has abundant coal bed methane (CBM) resources. CBM reserves buried lower than 2000 m are estimated to be 36.8 Tm^3^, accounting for 13% of the world's resources and ranking third in the world [72].

Coal seams provide one of the most attractive sites for CO_2_ geological sequestration in China as a result of the huge resources and the high and stable adsorption of CO_2_, particularly in combination with ECBM [26, 73, 74]. Adsorption is the main trapping mechanism for CO_2_ storage in coal seams, which accounts for approximately 90% of the total storage. The ECBM potential associated with CO_2_ sequestration is estimated to be over 3.751 × 10^12^ m^3^. And the CO_2_ sequestration capacity of China coal beds is estimated to be about 142.67 × 10^9^ t [75]. Based on the assessment for coal beds of China in depth between 300 m and 1500 m, 1.632 × 10^12^ m^3^ methane can be increased from CO_2_-ECBM, and about 12.078 × 10^9^ t of CO_2_ can be stored [26].

The Yaojie coalfield is located in the western margin of Minhe and extends across the Gansu and Qinghai provinces of China. The Haishiwan coalfield is located in the deep part of the Yaojie coalfield. High concentrations of CO_2_ (34.1–98.64%) have been observed in the number 2 coal seam of Haishiwan coalfield [69].

**Figure 8:**
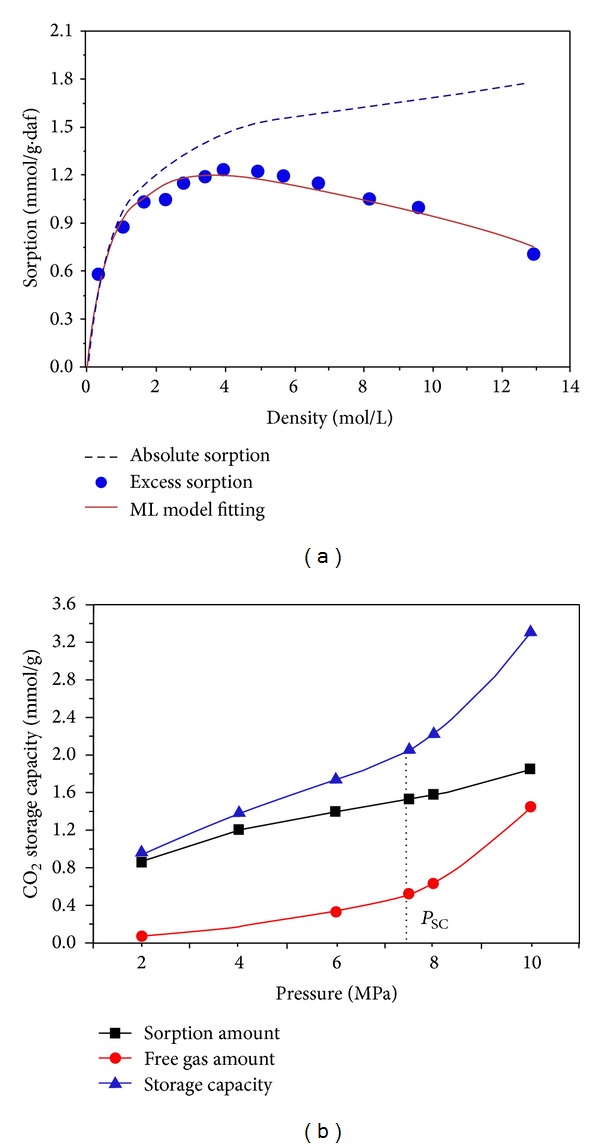
High-pressure CO_2_ adsorption on the dry Haishiwan coals at 40°C with respect to density (a); CO_2_ excess sorption isotherms and free CO_2_ content versus pressure (b); *P*_SC_ is the critical pressure of CO_2_ (from Li et al. [69]).

And the temperature-pressure conditions in Haishiwan coalfield indicate that supercritical CO_2_ may occur in the eastern half of the coalfield. Moreover, the Haishiwan coalfield is an ideal storage area because of the good sealing features and the presence of large volumes of juvenile CO_2_ that have been naturally sequestered over 15 million years. The pure CO_2_ storage capacity of the Haishiwan coal seam is 44.7 m^3^/t at 7.5 MPa and 313.15 K [69], as shown in Figure 8.

### 3.3. Other Ways of Sequestration

Plantation forests are the most effective and ecofriendly way of absorbing CO_2_ and increasing carbon sinks in terrestrial ecosystems, mitigating global warming and promoting ecological restoration. China's forestation rate is the highest in the world, contributing significantly to the nation's carbon sequestration [76]. Cost of carbon mitigation through plantation is relatively low, generally under $10/t, compared with $25–120/t for cost limitation of energy industry [77].

China currently has one of the world's most ambitious reforestation and afforestation programs, known as grain for green, which has been in place since 1999. It gives grain payouts to farmers who convert fields to forests. It is operating in many different regions across China. Although not one of its goals, carbon sequestration is a cobenefit of the program [78].

From 1950 to the present, plantations in China sequestered 1.686 PgC by net uptake into biomass and emission of soil organic carbon. Huang et al. [76] projected that China's forestation activities will continue to net sequester carbon to a level of 3.169 PgC by 2050.

On the other hand, China's rice paddies, accounting for 19% of the world's total, play an important role in soil carbon sequestration. The simulations demonstrated that all the recommended management practices could result in an increase in carbon sequestration potential, varying greatly from 29.2 to 847.7 TgC by 2050 [79].

Additionally, CH_4_–CO_2_ reforming can effectively convert CO_2_ and CH_4_ into synthesis gas. Interests regarding the CO_2_ reforming of CH_4_ have been rising due to the feasible approach for resource utilization and greenhouse gas emission reduction and the generated raw materials needed by many manufacturing process. Many efforts have been carried to devote and investigate various types of catalysts to promote the conversion process [80–84].

Overall, Table 1 summarizes the main comparative information of the above CO_2_ sequestration projects regarding different storage ways.

## 4. Challenge for Future

CCS is somehow a quite new technology in China. Even though a lot of assessments and potential analysis have been carried out across China, the real commercial implementations are limited. Various factors are supposed to be taken into consideration to promote CO_2_ sequestration and to mitigate the deteriorating environment in China.

International engagement is critical in developing and enlarging CO_2_ sequestration. China has already cooperated with other countries to start up a number of projects regarding CCS in many fields. However, more combined efforts are needed to move forward.

Technology is the priority determinant in CCS operation, including the technique from capture, transportation, assessment, and storage. The main oilfields in China are manifested in complex formation structure with strong heterogeneity, low or ultralow permeability, low porosity, and poor oil property [1]. CO_2_-EOR techniques would be challenged by high miscible pressure, severe gas channeling, heavy solid deposition, and development of complex reservoir [85].

On the other hand, effective policies are suggested to encourage and boost the CCS industry in China. Alternative ways should be developed to capture CO_2_ and reduce CO_2_ emission for different emitters.

Carbon emission trading system is forming in China. Market mechanism is important to reduce carbon emissions for China's low-carbon future [86].

## 5. Conclusion

The demand for clean energy and low-carbon technologies is enormous in China, where the rapid growth and heavy reliance on coal provide a mass of opportunities for application of new techniques. A great amount of CO_2_ can be sequestered by geological media, forestation, soil, and reforming. As a result, CCS is the most attractive way for reducing CO_2_ emission in China.

CO_2_ sequestration in depleted oil and gas reservoirs, saline aquifers, and coal beds is promising in China. A great number of projects have been implemented to testify the feasibility of CCS, examine the potential for commercial-scale CCS, and assess the storage capacity and possibility of CSEOR in large parts of China like Jilin oilfield, the first large-scale demonstration project on CSEOR.

Forestation, soil, and CO_2_ reform could provide alternative ways for CO_2_ sequestration. Combination of variety of methods can deeply promote the emission-reducing work.

There is a gap in carbon sequestration between China and other countries. Besides, most of the CO_2_ storage projects in China are still in the evaluation and assessment stage. Further efforts are needed to move forward, involving international cooperation, advanced technology, positive policy, and society mechanism.

## Figures and Tables

**Table 1:** Comparison of different CO_2_ sequestration projects.

Storage media	Total CO_2_ storage capacity	Project	CO_2_ storage capacity	EOR potential	Cost of storage
Oilfield	4.6 × 10^9^ t (>800 m)	Jilin	0.7 × 10^6^ t	0.5 × 10^6^ t	1 : 1.37 (input : output)
		Caoshe	0.309 × 10^9^ m^3^ (by 2009)	0.03 × 10^6^ t (by 2009)	$25.78/t
		Jingbian	0.04 × 10^6^ t/yr	0.05 × 10^6^ t/yr	
		Changqing	0.098 × 10^9^ t	0.239 × 10^9^ t	
		Shengli	95.539 × 10^6^ t	9.997 × 10^6^ t	
		Xinjiang	0.495 × 10^9^ t		

Gas field	30.483 × 10^9^ t (900–3500 m)	DF1-1	0.511 × 10^9^ Sm^3^		$20/t

Saline aquifer	143.505 × 10^9^ t	LT13-1	0.1 × 10^9^ t		$33–37/t
		Bohai Bay	3.9 × 10^9^ t		
		Songliao	8.96 × 10^9^ t		

Coal seam	142.67 × 10^9^ t	Haishiwan	44.7 m^3^/t		

Plantation	3.169 PgC (by 2050)				<$10/t

Soil carbon sequestration	29.2–847.7 TgC (by 2050)				
